# Oestrogen receptor phosphorylation profiles and *in silico* PAM50 subtyping reflect sexual dimorphism in breast cancer

**DOI:** 10.1002/2056-4538.70101

**Published:** 2026-06-23

**Authors:** Subarnarekha Chatterji, Aimee Diack, Marcin Szostok, Michael D Morgan, Valentina Silvestri, Laura Ottini, Cathy B Moelans, Paul J van Diest, Cigdem Selli, Andrew H Sims, Rasha Abu‐Eid, Valerie Speirs

**Affiliations:** ^1^ School of Medicine, Medical Sciences and Nutrition University of Aberdeen Aberdeen UK; ^2^ Aberdeen Cancer Centre University of Aberdeen Aberdeen UK; ^3^ Department of Molecular Medicine Sapienza University of Rome Rome Italy; ^4^ Department of Pathology University Medical Center Utrecht Utrecht The Netherlands; ^5^ Cancer Research UK Scotland Centre (Edinburgh), Institute of Genetics and Cancer University of Edinburgh Edinburgh UK; ^6^ School of Dentistry, School of Health Sciences, College of Medicine and Health University of Birmingham Birmingham UK; ^7^ Present address: Integrated Pathology Unit The Institute of Cancer Research and Royal Marsden NHS Foundation Trust London UK; ^8^ Present address: University Hospitals Sussex NHS Foundation Trust, Worthing Hospital West Sussex UK

**Keywords:** breast cancer, sex, genomics, oestrogen receptor, phosphorylation

## Abstract

Breast cancer (BC) is most prevalent in females but also accounts for <1% of male cancer cases and 0.2% of male cancer‐related deaths. Distribution of histological subtypes, receptor status, and age of diagnosis varies based on sex, and a growing body of evidence supports sex‐specific molecular differences in BC. However, this is limited by the smaller number of male cases available for study compared to the thousands of cases of female BC. We combined publicly available male BC gene expression datasets for 195 patients from 4 studies and split randomly into discovery and validation sets. Clustering and gene expression analysis were performed. Two stable clusters were identified initially, confirmed in the validation set. Cluster C1 was enriched for genes associated with MAPK signalling and arylesterase activity. Cluster C2 showed enrichment of genes associated with proliferation, invasion, and metastasis, along with enrichment of gene ontology and pathway terms related to ECM regulation, particularly collagen‐containing ECM. Of note, when stratified by ERα and PR status, no enrichment was observed with the predicted PAM50 classification. ERα and MAPK signalling were enriched in both clusters, albeit through different gene sets. Since these pathways were enriched, we investigated the signalling regulation of ERα based on immunohistochemical expression of phosphorylated ERα (S104, S118, S167, S294) and their prognostic roles. This analysis also revealed distinctions from female BC, showing a lack of prognostic outcome for any of these biomarkers. We show that male BC does not align with female BC in the same way that intrinsic subtypes of female BC are not identical. As BC heterogeneity is well recognised, we propose that male BC should be considered as a potentially unique clinical subtype of BC.

## Introduction

Sexual dimorphism is increasingly recognised in biology and has been reported in cancer, with breast cancer (BC) showing the biggest dichotomy [1]. While BC is still rare in males, the numbers receiving a BC diagnosis have increased globally from 8,500 diagnoses in 1990 to 23,100 in 2017, with age‐standardised rates in 100,000 person years of 0.46 and 0.61, respectively [2]. There is evidence that increased obesity aligns with this increase [3]. That we are an ageing population may also contribute, particularly to reduced overall survival (OS), but this does not account for the lack of improvement in BC‐specific survival observed for men diagnosed with stage III or IV BC over the past 30 years [4].

A striking feature of male BC is its almost universal positivity for both oestrogen receptors (ER) ‐α and ‐β [5, 6]. ERα, used to determine clinical outcome in female BC, is expressed in almost 90% of all male BCs in contrast to the 60–70% expression observed in females and confirmed in two of the largest studies on male BC [6, 7]. Male BC is thus considered, somewhat counterintuitively, to be ER driven with the most recent ASCO guidelines advocating tamoxifen as first line endocrine therapy [8]. When bound to ligands like tamoxifen, ERα becomes phosphorylated. Many phosphorylation sites exist on ERα and S104, S118, S167, S282, S294, T311, and S559 are the best characterised in female BC [9]. The impact of ERα phosphorylation in male BC is unknown.

Reflective of its propensity for high ERα‐positivity, male BC is predominantly of Luminal phenotype [6, 10]. This is a well‐recognised intrinsic subtype of female BC [11] and from this, PAM50, a 50‐gene subtype predictor, including ERα, PR (progesterone receptor), and HER2 (human epidermal growth factor receptor 2), was developed [12]. Marketed as Prosigna™, the commercial PAM50 assay can estimate the risk of future recurrence in hormone receptor‐positive female BC [13]. Further stratification of female BC into 10 subgroups has been achieved by the METABRIC group [14]. Similar attempts have been made to identify specific subtypes of male BC. Comparative genomic hybridisation (CGH) of tumour DNA from 56 cases applied to a BAC array identified male‐simple and male‐complex stratification. Cases in the smaller male‐simple cluster were associated with better prognosis: small size, reduced S‐phase and fewer genomic alterations while male‐complex aligned closely with the Luminal B subtype identified in females [15]. Two subgroups were identified in the same cohort of male BC patients using transcriptomics and an unsupervised clustering approach: Luminal M1 and M2 which were not represented by the intrinsic subgroups of female BC [16]. Combining the CGH and expression data derived from these two studies identified *TAF4* and *CD164* as the only two genes which were common between male and female BC, indicating that candidate driver genes of BC are not the same between sexes [17]. Two clusters were also observed through whole transcriptome analysis of male BCs with germline mutations in the most relevant BC susceptibility genes (*BRCA1/2, PALB2*, *RAD50*, *RAD51D*) with higher expression of immune response genes and high scores of gene‐expression signatures associated with aggressive phenotype and reduced survival [18]. Other groups have also shown sex‐specific differences with reduced frequency of 16q copy number in male compared to female BC [19], and less frequent promoter hypermethylation [20]. While informative, these studies were limited in terms of case numbers, a common issue when studying male BC.

The aim of this study was twofold. First, we employed an integrated bioinformatics approach using published bulk RNA‐seq datasets to agnostically investigate the intrinsic subtypes of male BC and their transcriptomic characteristics. Second, based on these findings, we used immunohistochemistry to examine the expression and prognostic value of ERα phosphorylated at S104, S118, S167, and S294 in male BC.

## Materials and methods

**Figure 1:**
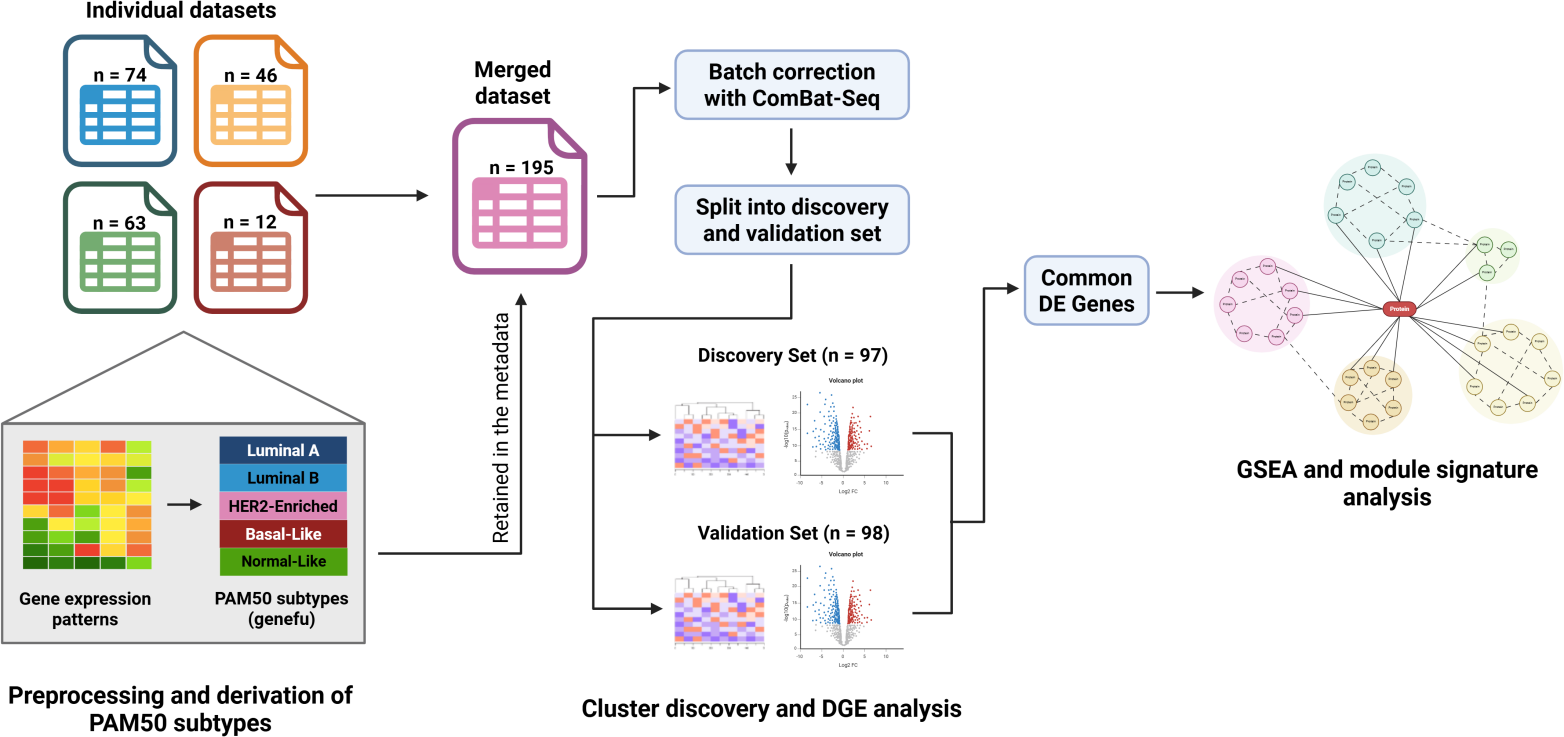
Experimental pipeline for cluster discovery. Following preprocessing, PAM50 gene signatures for each case were derived from individual datasets which were retained in the metadata. These datasets were merged, batch‐adjusted, and split randomly into discovery (*n* = 97) and validation (*n* = 98) sets. Cluster discovery and differential gene expression (DGE) analysis were performed separately on the datasets. GEX module signature score calculation and GSEA were performed on common differentially expressed genes in the discovery and validation sets. Created in BioRender. Chatterji, S. (2026). https://BioRender.com/ujqg65o

A pipeline of the integrated bioinformatics approach is shown in Figure 1 with specific details presented below.

### Dataset selection and pre‐processing

Datasets were identified from The Cancer Genome Atlas (TCGA) [21], National Centre for Biotechnology Information (NCBI) – Gene Expression Omnibus (GEO) [22], and ArrayExpress [23], and by contacting authors of previously published studies [18, 24]. Five non‐RNA‐seq high throughput datasets were excluded [17, 25, 26, 27, 28] with only bulk RNA‐seq datasets sequenced on Illumina platforms included to minimise technical bias. Details of the datasets used are provided (supplementary material, Table S1). Sample identifiers for each patient included in datasets obtained from NCBI‐GEO and TCGA are given in supplementary material, Table S2. From this, 195 cases were suitable for analysis. For consistency, all were mapped to Entrez gene IDs using the biomaRt package [29, 30] in R. In case of multiple mappings to the same gene ID, the averages of the raw counts were used. When data were available in the form of FASTQ files, alignment to the reference human genome (Ensembl GRCh38.p14) with STAR v2.7.11 [31] and raw counts were obtained using HTSeq v2.0.4 [32]. These steps were performed using Python 3.11. For visualisation, HUGO Gene Nomenclature Committee (HGNC) gene symbols were used. Manual pre‐processing of each dataset was done separately to account for the differences in the formats of the available processed data as detailed in Supplementary Materials and Methods. PAM50 intrinsic subtypes were determined by applying the *genefu* package [33] to each dataset separately. The resulting subtypes were retained in the clinical information associated with each patient for further analysis.

### Integration, batch correction, and patient randomisation

Pre‐processed datasets were combined using the merge function in R. Only those genes that were common to all datasets were included for further analysis (*n* = 15,674). Since datasets were combined from multiple sources, it was necessary to minimise batch effects due to technical differences while retaining the biological signals. For batch correction, the ComBat‐seq method [34] available from the sva package in R [35] was applied to the filtered read counts. Batch correction was confirmed by principal component analysis (PCA) both before and after application of ComBat‐seq (supplementary material, Figure S1). The batch adjusted dataset was randomly sampled using the sample function in R into discovery (*n* = 97) and validation sets (*n* = 98). Subsequent analyses were performed independently on these datasets.

### Unsupervised hierarchical clustering and differential gene expression (DGE) analysis

Generation of gene expression matrices and subsequent DGE analysis for the discovery and validation sets were performed with the DESeq2 package [36]. The variance stabilising transformation method was used to normalise gene expression matrices using the vst package [37]. Subgroups with common gene expression patterns were then estimated based on the 2000 most variable transcripts in each dataset using unsupervised hierarchical clustering following Ward's minimum variance method [38]. The top 2,000 variable genes were analysed with the assumption that these probably represented the major sources of variation in the data, in accordance with other BC studies [39, 40]. Stability of the resulting clusters was evaluated by multiscale bootstrap resampling using pvclust [41], where the number of bootstrapped datasets was set to 10,000, as per package recommendation to minimise error. Veracity of the estimated clusters was confirmed using clusterpval [42].

Differential expression between the clusters was determined based on the log2 (fold change) ≤1.0 (downregulation) and ≥1.0 (upregulation), with an adjusted *p* value of <0.05. False discovery rate was controlled for using the Benjamini Hochberg method.

### Gene expression (GEX) module signatures

GEX signatures associated with genes involved in proliferation (*AURKA*), apoptosis (*CASP3*), ERα signalling (*ESR1*), HER2 signalling (*ERBB2*), invasion and metastasis (*PLAU*), immune response (*STAT1*), and angiogenesis (*VEGF*) were calculated for each patient as follows:

$$\text{Module Score}=\frac{\Sigma_{i}w_{i}x_{i}}{\Sigma_{i}|w_{i}|}$$

For each gene 𝑖, its expression was denoted as 𝑥, while the value of 𝑤 was +1 or −1 depending on whether gene 𝑖 was up or downregulated in the female BC study that defined these signatures [43]. These module scores were calculated for both the discovery and validation sets, and the difference in the scores between clusters was determined using the Wilcoxon rank‐sum test.

### Gene set enrichment analysis (GSEA)

GSEA was performed using Enrichr [44] to determine the biological relevance of differentially expressed genes (DEGs) between the clusters. This was applied to DEGs that were common to discovery and validation sets. Gene ontology (GO) enrichment analysis was performed based on biological process (BP), cellular component (CC), and molecular function (MF) [45]. Pathway analysis was performed by interrogating the Reactome Pathway database [46], the National Centre for Advancing Translational Sciences (NCATS) BioPlanet database [47], the Kyoto Encyclopaedia of Genes and Genomes (KEGG) [48], WikiPathways [49], and Molecular Signatures Database (MSigDb) Hallmarks [50]. Significance of the key terms from each database was determined using Benjamini Hochberg FDR‐adjusted *p* values of <0.05. Enrichment scores were calculated as the −log10 of the adjusted *p* value. The key terms were sorted according to decreasing enrichment scores and the top 10 terms from each database that reached statistical significance were reported for each cluster.

### Immunohistochemistry (IHC)

A cohort of 506 male BCs was represented on tissue microarrays (TMAs) as described previously [28]. Three separate cores were sampled from different areas of each tumour case and placed into TMAs. Clinical characteristics are shown in supplementary material, Table S3. Ethical approval was obtained from the Leeds (West) Research Ethics Committee (06/Q125/156), Greater Glasgow Health Board (Network Approval: TR000269), and the NHS Grampian Tissue Bank Committee (Network Approval: TR000269). Four phosphorylated ER epitopes, which had been validated previously in female BC [9] were detected immunohistochemically (Leica BOND III Protocol F; BOND Refine Detection Kit). Details of antibodies used are provided in supplementary material, Table S4.

Following IHC, TMA slides were scanned (ZEISS AxioScan Z1 slide scanner 20×) generating whole slide images (WSIs). These were visualised using QuPath [51] open‐source image analysis software (v0.4.4510), which was also used for all subsequent image analysis steps, including stain vector normalisation, TMA dearraying, tissue detection, annotation of tumour and stromal regions, and stain quantification. The Allred system was used for nuclear staining and percentage of positive tumour cells for cytoplasmic staining. Scoring and assessment was done blinded to patient characteristics and outcome.

### Statistics

All statistical analyses and data visualisation were performed in R 4.3.0. OS was defined as the time between diagnosis to death from any cause or the date of last follow‐up. Log‐rank tests and Cox proportional hazards models based on 5‐ and 10‐year OS were used for prognostic assessment. Correlation between analytical and clinicopathological variables was assessed using Fisher's exact test with the Bonferroni method to correct for multiple testing. A *p* value <0.05 after adjustment for multiple testing was considered significant.

## Results

### Unsupervised hierarchical clustering reveals two stable clusters in male BC

Two stable clusters were identified in the discovery and validation sets (supplementary material, Figure S2), termed Cluster C1 and C2. We tested the null hypothesis that there was no difference in gene expression levels between the two clusters of patients. For both our discovery and validation datasets, we identified numerous significant gene expression differences between Clusters C1 and C2 (*p* < 0.001). To rule out potential confounding from specific studies in our meta‐analysis, we examined whether cluster separation in either the discovery or validation data was driven by any of the source datasets by Fisher's exact test between the clusters and the source datasets. No such evidence was found as no significant correlation was seen between these with any one cluster in either the discovery or validation sets (*p* = 0.07; *p* = 0.269) (supplementary material, Table S5). Cluster C1 was smaller in both sets: discovery set (*n* = 14; approximately unbiased [AU] probability = 76%), validation set (*n* = 22, AU probability = 85%). Cluster C2 had 83 patients in the discovery set and 76 in the validation set (AU probabilities 77%, 86%, respectively). These checks confirmed data fidelity.

### Differential gene expression (DGE) analysis

**Figure 2:**
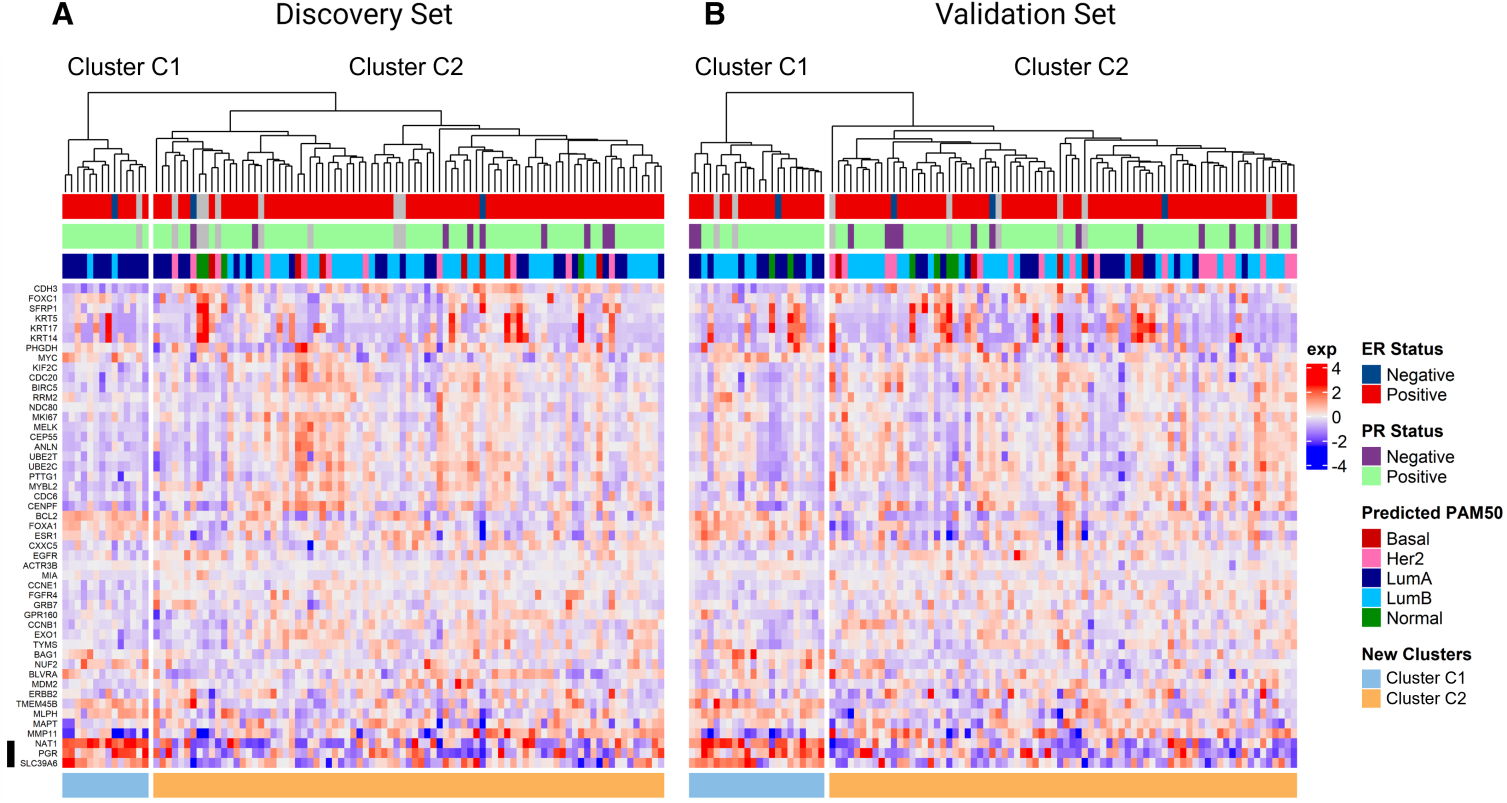
Expression of the 604 common DEGs in the discovery and validation sets and their relationship with PAM50. Heatmaps generated from the discovery (A) and validation (B) sets. Hormone receptor status and predicted PAM50 phenotype are reported for each case. *SLC39A6*, *NAT1*, and *PGR* were upregulated in Cluster C1 of both the discovery and validation sets (black vertical line).

DGE was analysed between Clusters C1 and C2 in the discovery and validation sets. This revealed 1,168 DEGs in the discovery set (409 upregulated and 759 downregulated genes in Cluster C1, with the opposite pattern of regulatory state seen in Cluster C2). In the validation set, 1,294 genes were differentially expressed (367 upregulated and 926 downregulated genes in Cluster C1, with Cluster C2 exhibiting the opposite pattern of regulation; supplementary material, Figure S3). There were 603 common differentially expressed genes between the discovery and validation data sets, of which 230 were downregulated and 373 were upregulated in Cluster C1. No significant difference in the mean log2 (fold change) was observed between the discovery and validation sets for the common genes (*p* = 0.24). Expression patterns of the 604 common DEGs in the discovery and validation sets are shown in Figure 2. Upregulation of *SLC39A6*, *NAT1*, and *PGR*, which showed the highest differential expression in both discovery and validation sets, was observed in Cluster C1.

Survival was assessed in a subset of the cohort that had complete OS information. This was available for 108 of 195 cases analysed. While cases from Cluster C2 had a trend towards poorer survival, no significant differences were observed between cases from Clusters C1 and C2 at either 5‐ (*p* = 0.08) or 10‐year OS (*p* = 0.29). This is shown in supplementary material, Figure S4.

To resolve whether the two clusters of male BC aligned with previous female BC subtypes, datasets were assessed further for enrichment of PAM50 features. Their enrichment in each cluster was examined with Fisher's exact test. Cluster C1 was significantly correlated with the Luminal A subtype in both discovery and validation sets (adjusted *p* = 0.009 and *p* = 0.01, respectively; supplementary material, Table S6). Interestingly, when stratified by ERα and PR status, no enrichment was observed with the predicted PAM50 classification in either dataset (supplementary material, Table S7).

### Gene set enrichment analysis (GSEA)

We applied GSEA to examine how predefined groups of genes were altered in male BC. GSEA scores and their associated genes are shown in Table 1. GO and pathway terms enriched in Cluster C1 included genes involved in basement membrane (GO: CC), oxidative stress response (WikiPathways), arylesterase activity (GO: MF), KRAS signalling activation and late oestrogen response (both MSigDb Hallmarks). MAPK signalling upregulation was seen in multiple platforms (NCATS BioPlanet, KEGG, WikiPathways). In Cluster C2, genes involved in extracellular matrix (ECM) organisation and regulation were upregulated. Terms relating to this included ECM‐receptor interaction (NCATS BioPlanet, KEGG), ECM organisation (GO: Biological Process, Reactome Pathway), collagen‐containing ECM (GO: CC), IL‐1 regulation of ECM and TGF‐β regulation of ECM (both NCATS BioPlanet). Genes involved in the Hedgehog (NCATS BioPlanet) and Hippo signalling pathways (KEGG), oncostatin M (NCATS BioPlanet), regulation of cell population proliferation (GO: Biological Process), hormone activity, receptor ligand activity, cytokine activity (all GO: MF), epithelial to mesenchymal transition, downregulated genes due to KRAS signalling activation, early oestrogen response (all MSigDb Hallmarks), and differentiation of white and brown adipocytes (WikiPathways) were also enriched in Cluster C2. Significantly enriched GO terms and pathways that had high gene counts are shown in Table 1.

**Table 1:** Enrichment terms and their associated genes, along with their regulation in Clusters 1 and 2, sorted in descending order of enrichment score

Term	Source	Regulation (Cluster C1)	Regulation (Cluster C2)	Count	Genes	FDR‐adjusted *p* value	Enrichment score
Collagen‐Containing Extracellular Matrix (GO:0062023)	GO: Cellular Component	Down	Up	31	*FBN2; SPON1; COL11A1; F13A1; DPT; AEBP1; THBS2; ORM2; COCH; COMP; HPX; CDH2; COL10A1; ANXA8; TPSAB1; WNT2; POSTN; RARRES2; KRT1; SULF1; NPNT; COL2A1; LOX; MMP23B; COL5A2; MFAP2; S100A9; FREM2; MATN3; S100A8; S100A7*	6.61e‐10*	9.18
Oncostatin M	NCATS BioPlanet 2019	Down	Up	22	*FBN2; EDN1; BEX1; CALML5; CRABP2; CCL21; C1S; IL1R1; ANXA3; KRT1; MMP3; KRT13; CST6; HOXA9; CDH3; SLC16A3; S100A9; CAMP; DSC2; S100A8; S100A7; MSMB*	5.5e‐05*	4.26
Epithelial Mesenchymal Transition	MSigDb Hallmarks	Down	Up	15	*FBN2; POSTN; NNMT; COL11A1; TFPI2; MMP3; THBS2; FSTL3; COMP; CDH2; LOX; DPYSL3; COL5A2; SDC1; MATN3*	7.8e‐05*	4.12
Oestrogen Response Early	MSigDb Hallmarks	Down	Up	15	*LAD1; CLIC3; NXT1; SLC24A3; ELOVL2; TMPRSS3; KRT13; INHBB; ADD3; SYNGR1; KRT15; P2RY2; PMAIP1; NBL1; MSMB*	7.8e‐05*	4.12
KRAS Signalling Down	MSigDb Hallmarks	Down	Up	15	*EDN1; EDN2; CALML5; KRT1; KRT13; YBX2; OXT; TCL1A; COL2A1; KRT15; PKP1; EFHD1; CACNG1; FGFR3; ATP6V1B1*	7.8e‐05*	4.12
Hormone Activity (GO:0005179)	GO: Molecular Function	Down	Up	10	*NPPC; PYY; EDN1; EDN2; VGF; LEP; PENK; OXT; INHBB; METRNL*	3.88e‐04*	3.41
Oestrogen Response Late	MSigDb Hallmarks	Up	Down	11	*SCUBE2; SERPINA1; CA2; IGSF1; SEMA3B; RBBP8; DNAJC12; CPE; PGR; PRSS23; FRK*	3.97e‐04*	3.40
Receptor Ligand Activity (GO:0048018)	GO: Molecular Function	Down	Up	20	*EDN1; EDN2; WNT3A; WNT7B; IL19; INHBB; METRNL; BMP7; SLURP1; BMP5; BMP4; NPPC; PYY; VGF; LEP; FAM3B; NRTN; NBL1; WNT2; WNT4*	4.49e‐04*	3.35
Cytokine Activity (GO:0005125)	GO: Molecular Function	Down	Up	14	*EDN1; CCL21; WNT3A; WNT7B; IL19; INHBB; BMP7; SLURP1; BMP5; BMP4; KLF5; FAM3B; WNT2; WNT4*	7.65e‐04*	3.12
KRAS Signalling up	MSigDb Hallmarks	Up	Down	10	*TPH1; KIF5C; CA2; SEMA3B; MAP7; CPE; GLRX; TSPAN1; ETV4; SCN1B*	1.38e‐03*	2.86
Differentiation of White and Brown Adipocyte (WP2895)	WikiPathways 2023	Down	Up	6	*BMP4; LEP; CIDEA; HOXC9; HOXC8; BMP7*	1.74e‐03*	2.76
Arylesterase activity	GO: Molecular Function	Up	Down	3	*PON3; CA2; PON1*	4.56e‐03*	2.34
Extracellular Matrix Organisation (RIA‐1474244)	Reactome Pathway Database 2022	Down	Up	18	*FBN2; COL11A1; MMP3; BMP7; COMP; BMP4; MMP11; COL2A1; CAPN12; LOX; COL5A2; ITGA11; MFAP2; SDC1; COL10A1; ITGB7; TPSAB1; MATN3*	6.41e‐03*	2.19
MAPK signalling pathway	KEGG 2021	Up	Down	12	*DUSP4; CACNB2; NTRK2; RPS6KA3; PDGFD; NTF3; CACNA2D3; MAP3K8; CACNA1D; PRKACB; MAP3K5; FGF10*	1.13e‐02*	1.95
Hedgehog signalling pathway	NCATS BioPlanet 2019	Down	Up	8	*BMP4; ZIC2; WNT3A; WNT7B; WNT2; BMP7; WNT4; BMP5*	1.24e‐02*	1.91
Hippo signalling pathway	KEGG 2021	Down	Up	12	*BMP4; WNT3A; WNT7B; FZD9; BIRC5; WTIP; WNT2; BMP7; TEAD2; WNT4; BMP5; NKD2*	1.26e‐02*	1.89
Basement Membrane (GO:0005604)	GO: Cellular Component	Up	Down	5	*SMOC2; SMOC1; LAMA3; COL4A6; VWA2*	1.39e‐02*	1.86
ECM‐receptor interaction	KEGG 2021	Down	Up	8	*COMP; COL2A1; ITGA11; SDC1; ITGB7; NPNT; THBS2; FREM2*	1.75e‐02*	1.76
Regulation Of Cell Population Proliferation (GO:0042127)	GO: Biological Process	Down	Up	30	*ATP8A2; UHRF1; CDCA7; SIX1; ADRA1D; HOXC10; FUT3; TCL1A; SCIN; SIX4; PDPN; PRAME; WNT2; SOX4; EDN1; EDN2; WNT3A; NCCRP1; ALOX15B; SLURP1; BMP5; BMP4; CLDN3; FABP6; BAMBI; BIRC5; FGFR4; FGFR3; TSPYL5; BNIPL*	2.42e‐02*	1.62
Interleukin‐1 regulation of extracellular matrix	NCATS BioPlanet 2019	Down	Up	9	*COMP; BMP4; MMP11; SCIN; COL2A1; LOX; MMP3; SAA2; MATN3*	2.39e‐02*	1.62
Epithelium Development (GO:0060429)	GO: Biological Process	Down	Up	11	*DLX3; LHX1; KRT37; WNT7B; KRT15; RIPK4; KRT13; SIX1; ALDOC; WNT2; WNT4*	2.43e‐02*	1.61
TGFβ regulation of extracellular matrix	NCATS BioPlanet 2019	Down	Up	22	*EDN1; POSTN; CRABP2; C1S; IL1R1; CCNF; IDH2; TFPI2; AEBP1; THBS2; CST6; BMP4; CSRP2; CDH2; LOX; BAMBI; RBP1; ITGA11; SAA2; TRIB3; PDE5A; MATN3*	4.07e‐02*	1.39

Significant enrichment terms with their associated genes are listed along with their up/downregulation status in Clusters C1 and C2.

### GEX module signature analysis

**Figure 3:**
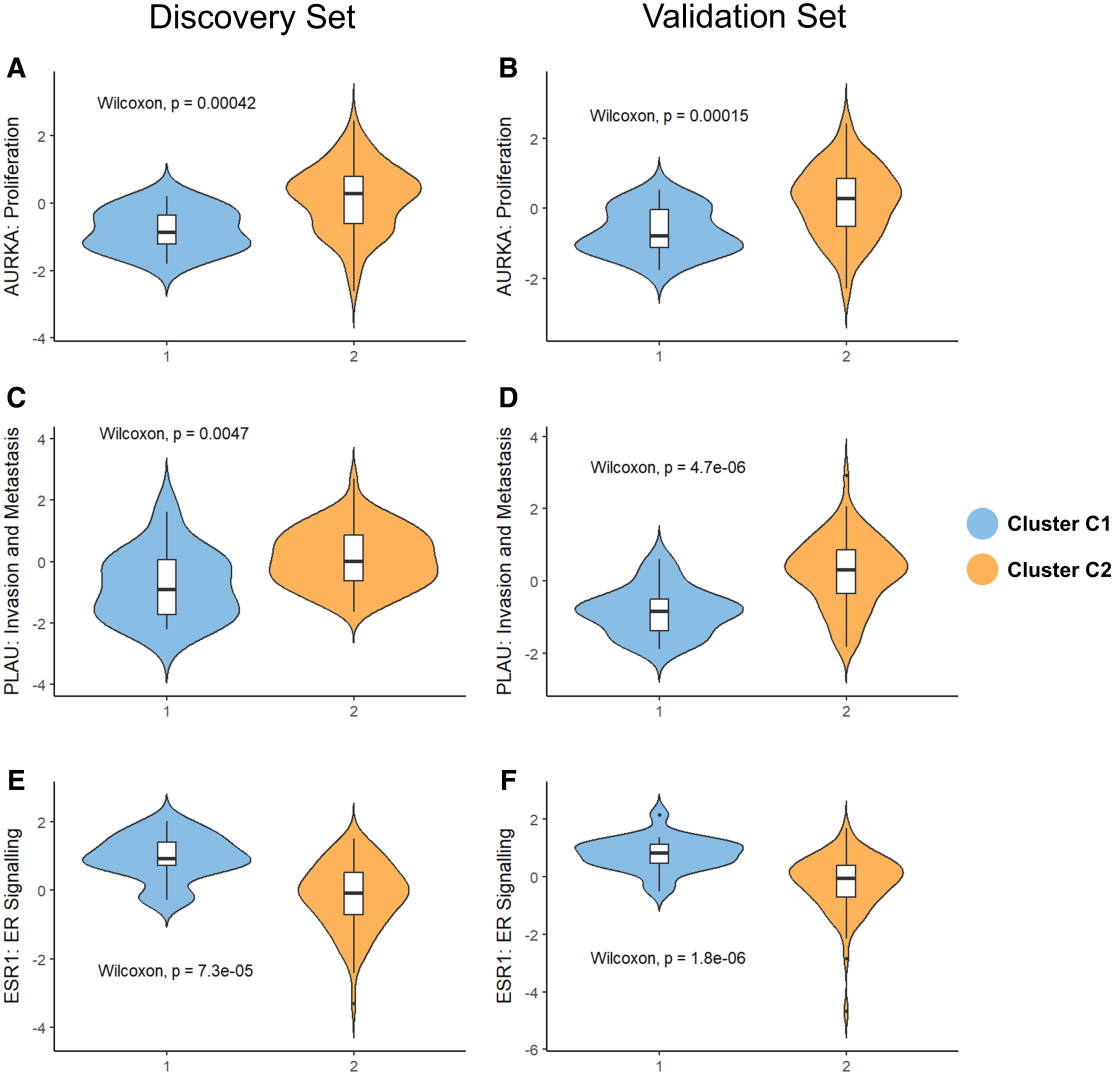
Distribution of GEX module scores that had significant differences between Clusters C1 and C2 in both discovery and validation sets. Violin plots showing the GEX modules that had significantly different scores between Clusters 1 and 2 in both the discovery and validation sets. These were (A and B) proliferation (*AURKA* signature), (C and D) invasion and metastasis (*PLAU* signature), and (E and F) ERα signalling (*ESR1* signature) modules.

To identify biological pathways involved in male BC, GEX module scores were calculated for each patient. These were compared between Clusters C1 and C2 for both discovery and validation sets. In both datasets, Cluster C1 had significantly higher scores for the ERα signalling (*ESR1* signature) module (*p* = 7.3e‐05 for discovery set; *p* = 1.8e‐06 for validation set). On the other hand, significantly higher scores for proliferation (*AURKA* signature) and invasion and metastasis (*PLAU* signature) modules were seen for Cluster C2 in both discovery (*p* = 4.2e‐04 and *p* = 4.7e‐03, respectively) and validation sets (*p* = 1.5e‐04 and *p* = 4.7e‐06, respectively). Violin plots showing the above distributions are given in Figure 3.

No other GEX modules examined showed significant differences between Clusters C1 and C2 consistently in both datasets. The HER2 signalling module (*ERBB2* signature) had higher scores in Cluster C2 (*p* = 0.03), but only in the discovery set. Similarly, the angiogenesis module (VEGF signature) had higher scores in Cluster C2 (*p* = 0.03), but only in the validation set. No differences were seen in the apoptosis (CASP3 signature) and immune response (STAT1 signature) modules between the clusters in either dataset. Violin plots showing the above comparisons are shown in supplementary material, Figure S5.

### Immunohistochemical expression and prognostic role of phosphorylated ERα

GSEA data showed upregulation of oestrogen response genes and MAPK signalling pathways. The MAPK pathway significantly influences ERα function, primarily through the phosphorylation of serine residues. Since the presence of ERα is always consistently higher in male than female BC, we reasoned that this may be due to ERα phosphorylation, a post‐translational modification that alters ERα activity and which has not been explored in male BC. While S104, S118, S167, and S294 all displayed nuclear and cytoplasmic staining (supplementary material, Figures S7–S9), only nuclear immunoreactivity, determined by the Allred system, was considered as this is its typical cellular location. Staining trends were comparable when manual scoring (Allred) was compared to QuPath‐generated values.

Due to missing data, clinicopathological information available was specific to each biomarker. Correlation between available clinicopathological variables and expression of each phosphorylated ERα was tested and is shown in Table 2. Low S104 expression was significantly associated with high tumour grade (*p* = 0.02), which lost significance after Bonferroni adjustment. No associations were seen for S118. Low nuclear expression of S167 was associated with PR and AR negativity (*p* = 0.02 and 0.002, respectively). High nuclear S294 correlated significantly with ERα and AR negativity (*p* = 0.03 and 0.04, respectively) and high tumour grade (*p* = 0.04), which was lost upon Bonferroni correction. No other significant associations were observed. Prognostic values were investigated using multivariate analysis (Table 3) for 5‐ and 10‐year OS; however, no significant associations were found.

**Table 2:** Clinicopathological associations of ERα phosphorylated at S104, S118, S167, and S294 according to cell location

Variable	Status	S104			S118			S167			S294		
		Low (*n*)	High (*n*)	Fisher's exact *p* value	Low (*n*)	High (*n*)	Fisher's exact *p* value	Low (*n*)	High (*n*)	Fisher's exact *p* value	Low (*n*)	High (*n*)	Fisher's exact *p* value
Age (years)	≤65	34	25	0.37 Adjusted *p* = 1.0	50	28	1.0 Adjusted *p* = 1.0	55	29	0.88 Adjusted *p* = 1.0	41	26	0.07 Adjusted *p* = 1.0
	>65	46	24		66	37		66	38		55	17	
Grade	1	7	5	0.02 Adjusted *p* = 0.44	13	6	0.32 Adjusted *p* = 1.0	14	8	0.1 Adjusted *p* = 1.0	8	6	0.04 Adjusted *p* = 1.0
	2	28	29		44	35		47	35		35	27	
	3	54	20		65	33		73	28		61	20	
Node status	+	43	25	0.7 Adjusted *p* = 1.0	62	25	0.1 Adjusted *p* = 0.1	60	27	0.62 Adjusted *p* = 1.0	54	20	0.69 Adjusted *p* = 1.0
	−	33	16		42	31		48	26		35	16	
ERα	+	91	68	1.0 Adjusted *p* = 1.0	132	74	1.0 Adjusted *p* = 1.0	139	77	0.17 Adjusted *p* = 1.0	102	67	0.03 Adjusted *p* = 0.6
	−	4	3		6	3		8	1		8	0	
PR	+	80	60	0.82 Adjusted *p* = 1.0	116	67	0.68 Adjusted *p* = 1.0	117	71	0.02 Adjusted *p* = 0.38	90	58	0.18 Adjusted *p* = 1.0
	−	14	9		20	9		28	5		19	6	
AR	+	63	50	0.16 Adjusted *p* = 1.0	81	39	0.19 Adjusted *p* = 1.0	73	49	0.002 Adjusted *p* = 0.06	72	50	0.04 Adjusted *p* = 0.9
	−	32	15		37	10		42	8		38	12	

AR, androgen receptor; ERα, oestrogen receptor alpha; PR, progesterone receptor.

**Table 3:** Multivariate analysis to assess the impact of ER phosphorylated at S104, S118, S167, and S294 on overall survival at 5 and 10 years

Phosphorylation site	5‐year OS				10‐year OS			
	HR	95% CI	*p*	Schoenfeld residual global *p* value	HR	95% CI	*p*	Schoenfeld residual global *p* value
S104	0.33	0.06–1.63	0.17	0.89	0.47	0.14–1.59	0.22	0.62
S118	0.86	0.38–1.98	0.73	0.73	0.80	0.40–1.62	0.54	0.29
S167	0.69	0.30–1.60	0.48	0.48	0.86	0.42–1.76	0.69	0.13
S294	0.78	0.20–2.98	0.6	0.6	0.86	0.28–2.65	0.8	0.39

ER, oestrogen receptor; OS, overall survival.

## Discussion

Molecular subtypes were first described in female BC some 25 years ago [11, 52] and have been gradually refined over the years. Transcriptomic profile comparisons between female and male BC have identified sex‐specific pathways related to ECM organisation, metabolism and protein translation [26, 28]. Incomplete overlap of hormone receptor signalling has also been reported [6, 24, 53]. More recently, 30 male and 54 female BCs were profiled in a 90‐gene expression PCR‐based assay which showed a distinct separation of sexes. Four genes (*PI15*, *AZGP1*, *PRRX1*, *AGR2*) were upregulated, and five (*PGR*, *SFRP1*, *PLA2G2A*, *S100A2*, *CHI3L1*) were downregulated in males, with the sex chromosomes *RPS4Y1 and XIST* up‐ and downregulated, respectively [54].

Attempts to stratify male BC using genomic approaches identified a two‐cluster profile by two independent research groups, with significant differences in prognosis and regulation of pathways relating to proliferation, invasion and metastasis observed [16, 18]. Since the current reporting framework for all BC is based on female BC and as sex‐specific discrepancies, notably the almost universal ER‐positivity in male BC, have been previously reported [6, 7], *a priori* stratification based solely on clinical features is challenging. Given the rarity of male BC, this warrants an agnostic approach. To address this, previously published bulk RNA‐seq datasets were combined, re‐split randomly into discovery and validation sets, with clustering and gene expression analysis performed independently on both. This approach also identified two clusters (C1 and C2) in both the discovery and validation sets, with Cluster C2 harbouring a more aggressive phenotype, in terms of proliferation, invasion and metastasis.

Cluster C2 was significantly enriched for GO and pathway terms related to ECM regulation and organisation, with collagen‐containing ECM showing highest enrichment. An immunohistochemical study of male BC showed that strong expression of collagen IV in the stroma predicted shorter disease‐free survival [55]. Deregulation of the collagen‐containing microenvironment has been reported in several cancers with p53 and JAK–STAT oncogenic pathways implicated [56, 57]. In pancreatic cancer, mutated *KRAS* proto‐oncogenes interacted with the EMT‐regulator Snail, resulting in enhanced collagen production [58]. Crosstalk between collagen and cancer cells can also be facilitated through TGF‐β/Smad signalling [59]. Interestingly, Cluster C2 was significantly enriched for genes downregulated due to KRAS signalling activation and those involved in EMT and TGF‐β mediated ECM regulation. It could be speculated that regulation of collagen‐containing ECM in some male BCs is similar to findings in pancreatic cancer where collagen production increased due to interactions between EMT‐regulators and *KRAS* [58, 60].

In the context of ECM, upregulation of genes involved in the Hippo signalling pathway in Cluster C2 is notable. This concurs with our previous findings that expression of TAZ and YAP, which are also Hippo transducers, along with their targets CTGF and AXL were predictors of poor OS in male BC [61, 62]. In agreement with this, a trend for poorer survival was observed in Cluster C2 for both 5‐ and 10‐year timepoints, which may potentially be significant in a larger and more balanced cohort. Thus, deregulation of collagen in the ECM may be an important mediator of male BC cancer progression. Detailed interrogation of the collagen‐containing ECM using surrogate biomarkers such as *COL2A1, COL4A1, COL11A1*, and *LOX* (all significantly upregulated in Cluster C2) alongside morphometric analysis could provide insight into potential functional roles. This is currently under investigation in our group.

We found enrichment of *ESR1* signature (ERα signalling module) in Cluster C1 and correlation with Luminal A phenotype. Intriguingly, no significant associations were found between hormone receptor status in male BC and the predicted PAM50 subtypes, which show strong correlation in FBC [13]. PAM50 has been applied in other male BC datasets, where a statistical association between immunohistochemical and PAM50 subtyping was seen [63, 64]. However, questions have been raised over the predictive ability of PAM50 in male BC since over half that were classified as Luminal A by immunohistochemistry were grouped differently by PAM50 [64]. It is noteworthy from these previous studies [63, 64] that PAM50 subtypes were derived using the Prosigna™ PAM50 assay [65], rather than using *genefu* predictions conducted in this work. This may question the concordance between computation and assay‐derived phenotypes. These challenges have been recognised in BC [66]. Lack of association between the PAM50 intrinsic subtypes and hormone receptor status from our *genefu* predictions may have arisen from the high imbalance in ERα/PR expression groups. However, this is unavoidable in male BC due to its almost universal ER‐positivity [6, 7]. It may also reflect the modest numbers of cases available. Nevertheless, our data showed that PAM50 used to stratify female BC does not apply in males.

While qualitative examination of the relative expression of the PAM50 genes in the Clusters C1 and C2 did not reveal any discernible patterns, consistent upregulation of *PGR*, *NAT1*, and *SLC39A6* was observed in Cluster C1. *NAT1* upregulation in Cluster C1 was consistent with previous findings [16, 18]. Differential expression of *PGR* did not match the consistent PR positivity across the study cohort, suggesting a non‐linear relationship between gene and protein expression.

GO terms related to oestrogen response were enriched in both Clusters C1 and C2, albeit through different gene sets. Cluster C1 was significantly enriched for late response pathways to 17β‐oestradiol (E2), while Cluster C2 was enriched for early response to E2. A meta‐analysis of oestrogen response in MCF‐7 cells showed that early response genes were related to cell growth and proliferation, while late response genes were related to cellular assembly and organisation, cell cycle, DNA replication, recombination and repair [67] – this was in agreement with our GEX module score findings as Cluster C2 was highly enriched for *AURKA* (proliferation) module score and exhibited a trend for poorer OS. This meta‐analysis also reported high regulation of the AhR (Aryl hydrocarbon Receptor) signalling pathway for both early and late response. The AhR pathway has been reported to inhibit ERα signalling in rat studies including direct inhibition by AhR/ARNT (Aryl hydrocarbon nuclear translocator) heterodimerisation, proteasomal degradation of ERα and increased CYP1A1 and CYP1B1 expression, inhibiting E2 synthesis [68]. Notably, Cluster C1 had significant enrichment for the GO term arylesterase activity. This may suggest increased inhibition of the ERα pathway in this cluster, with increased expression of the hormone receptor as a compensatory response.

We reasoned that despite overwhelming ERα positivity in male BC, the signalling pathway itself may be deregulated. Noting that our GSEA data showed upregulation of oestrogen response genes and MAPK signalling pathways in both clusters we hypothesised that this might impact on ER‐phosphorylation. Phosphorylation is fundamental in regulating steroid hormone receptor activity. ERα is phosphorylated at multiple sites, governed by kinase activity and phosphorylation at S104, S106, and S118 contributes to ERα activity [69]. ERα phosphorylation at S118 characterises an intact oestrogen‐dependent signalling pathway in BC and is associated with a better clinical outcome in female patients treated with tamoxifen [70, 71]. Also in female patients, ERα phosphorylation at S167 predicts significantly longer survival, particularly after endocrine relapse [72, 73]. While ER phosphorylated at S104, S118, S167 was detected in male BC, this did not impact on survival, contrasting the findings in female BC reported above. The association of low S104 expression that we observed with high tumour grade in male BC has been reported in female BC but was weaker [74] Alongside our deep learning work which showed that attention‐based machine‐learning trained on WSIs of female BC could accurately predict ERα status from a validation set of H&E‐stained WSIs of female BC but had poor discriminatory power when applied to male BC datasets prepared identically [75], these findings pose the intriguing possibility that ER function in BC may be sex‐specific. Indeed, sex specific features of ERα action have been identified in a genomic study. While most ERα binding sites were shared between male and female BCs in chromatin immunoprecipitation analysis, those associated with clinical outcome appeared sex specific [24]. ERα phosphorylation sites that can classify female outcome did not show predictive potential in male.

Limitations are acknowledged. The integrative bioinformatics focused solely on the genes common to all datasets. This was necessary to achieve data integration and minimise bias, but it leaves a potential gap *via* information loss through mutually exclusive genes. Like most studies on male BC, our results are limited by the number of patients available to analyse. These are small in comparison to similar work on female BC, but this is a common and well recognised obstacle when studying a rare pathology like male BC. Nevertheless, scope remains to validate these findings in larger datasets with comprehensive metadata. This was a retrospective study, which permitted accumulation of many more cases, which is an important consideration for a rare pathology. However, this meant that cases were identified and collected from a range of different sources, resulting in incomplete metadata for some cases as not all were systematically collected. However, scope remains to validate these findings in larger retrospective datasets such as the International Male Breast Cancer Program [7].

In conclusion, from the data presented herein, male BC is different biologically, in the same way that intrinsic subtypes of female BC are not identical. As BC heterogeneity is well recognised, we propose that male BC should not be separated from female BC but instead considered as a distinct and potentially unique subtype of BC. If verified mechanistically, the translational implications could potentially reshape how male BC is managed.

## Author contributions statement

VSp and AHS conceived and designed the study. SC and RA‐E contributed to the study design. SC, RA‐E and VSp contributed to data analysis, interpretation and writing of the manuscript. SC, AD, MS, RA‐E and VSp contributed to immunohistochemical analysis and interpretation. SC, VSi, LO, CBM and PJvD contributed to data collection and interpretation. Collection, analyses and interpretation of mRNA gene expression datasets were performed by SC, MDM, VSi and CS. Co‐authors gave critical input, and all authors read and approved the final submitted version of the paper.

## Supporting information


**Supplementary materials and methods.** Manual preprocessing of individual datasets
**Figure S1.** Confirmation of batch correction by principal component analysis (PCA)
**Figure S2.** Identification of two stable clusters in the discovery and validation sets
**Figure S3.** Volcano plots of DEGs, Clusters C1 and C2: discovery and validation sets
**Figure S4.** Kaplan–Meier curves, OS: cases in Clusters C1 and C2
**Figure S5.** Distribution of GEX module scores: similar or dissimilar results
**Figure S6.** Staining characteristics of S104
**Figure S7.** Staining characteristics of S118
**Figure S8.** Staining characteristics of S167
**Figure S9.** Staining characteristics of S294
**Table S1.** Male BC datasets used for integrated bioinformatics
**Table S2.** Sample identifiers for each patient: NCBI GEO and TCGA
**Table S3.** Clinical characteristics of patient cohorts
**Table S4.** Details of phosphorylated antibodies
**Table S5.** Breakdown of cases based on source dataset and their association with Clusters C1 and C2: discovery and validation sets
**Table S6.** Breakdown of cases based on predicted PAM50 subtype and the association with Clusters C1 and C2: discovery and validation sets
**Table S7.** Breakdown of cases based on their hormone receptor profiles and the estimated PAM50 subtypes: discovery and validation sets

## Data Availability

The public gene expression datasets are available from NCBI‐GEO GSE31259 and NCBI‐GEO GSE104730. Raw counts data in the TCGA‐BRCA dataset were downloaded individually from the Genomic Data Commons Data Portal and combined into a dataset. Other data that support the findings of this study are available from the corresponding author upon reasonable request.
