# Supplementary material for: Oestrogen receptor phosphorylation profiles and in silico PAM50 subtyping reflect sexual dimorphism in breast cancer
Source: J Pathol Clin Res. 2026 Jun 23;12(4):e70101. doi: 10.1002/2056-4538.70101 (PMC13288154; doi:10.1002/2056-4538.70101)
Supplement: Supplementary file 1 — Supplementary materials and methods. Manual preprocessing of individual datasets Figure S1. Confirmation of batch correction by principal component analysis (PCA) Figure S2. Identification of two stable clusters in the discovery and validation sets Figure S3. Volcano plots of DEGs, Clusters C1 and C2: discovery and validation sets Figure S4. Kaplan–Meier curves, OS: cases in Clusters C1 and C2 Figure S5. Distribution of GEX module scores: similar or dissimilar results Figure S6. Staining characteristics of S104 Figure S7. Staining characteristics of S118 Figure S8. Staining characteristics of S167 Figure S9. Staining characteristics of S294 Table S1. Male BC datasets used for integrated bioinformatics Table S2. Sample identifiers for each patient: NCBI GEO and TCGA Table S3. Clinical characteristics of patient cohorts Table S4. Details of phosphorylated antibodies Table S5. Breakdown of cases based on source dataset and their association with Clusters C1 and C2: discovery and validation sets Table S6. Breakdown of cases based on predicted PAM50 subtype and the association with Clusters C1 and C2: discovery and validation sets Table S7. Breakdown of cases based on their hormone receptor profiles and the estimated PAM50 subtypes: discovery and validation sets [file CJP2-12-e70101-s001.docx]

**Oestrogen receptor phosphorylation profiles and *in silico* PAM50 subtyping reflect sexual dimorphism in breast cancer**

S Chatterji *et al*. *J Pathol Clin Res* <https://doi.org/10.1002/2056-4538.70101>

**Supplementary materials and methods**

**Supplementary Figures S1–S9**

**Supplementary Tables S1–S7**

**Supplementary materials and methods**

**Manual preprocessing of individual datasets**

Preprocessing of each individual dataset was performed individually based on the format they were available in. The Johansson *et al* dataset (16; *n* = 74) was available from the NCBI-GEO (accession no. GSE31259) in the log2 transformed format.

The raw count data was obtained by reversing the log2 transformation. The processed Severson *et al* dataset ([24]; *n* = 46) was available on NCBI-GEO (accession no. GSE104730) in the rlog transformed format, which made it unsuitable for direct integration with the raw count data of the other datasets. To circumvent this, the FASTQ files for each patient were downloaded and aligned to the reference human genome (Ensembl GRCh38.p14) with STAR (v2.7.11) [31] using default parameters. Following alignment, raw counts were obtained using HTSeq (2.0.4) [32]. The above steps were carried out in Python 3.11.

The Zelli *et al* dataset ([18]; *n* = 63) in the form of raw counts and the associated clinical information was kindly provided by Professor Laura Ottini, Sapienza University of Rome, Italy. Further pre-processing was not required for this dataset.

Finally, the raw count data for each male patient (*n* = 12) in The Cancer Genome Atlas – Breast Cancer (TCGA-BRCA) dataset was downloaded individually from the [Genomic Data Commons (GDC) Data Portal](https://portal.gdc.cancer.gov/) and then combined into a dataset. The accompanying clinical information was downloaded from [cBioPortal for Cancer Genomics](https://www.cbioportal.org/).

**Supplementary Figures**


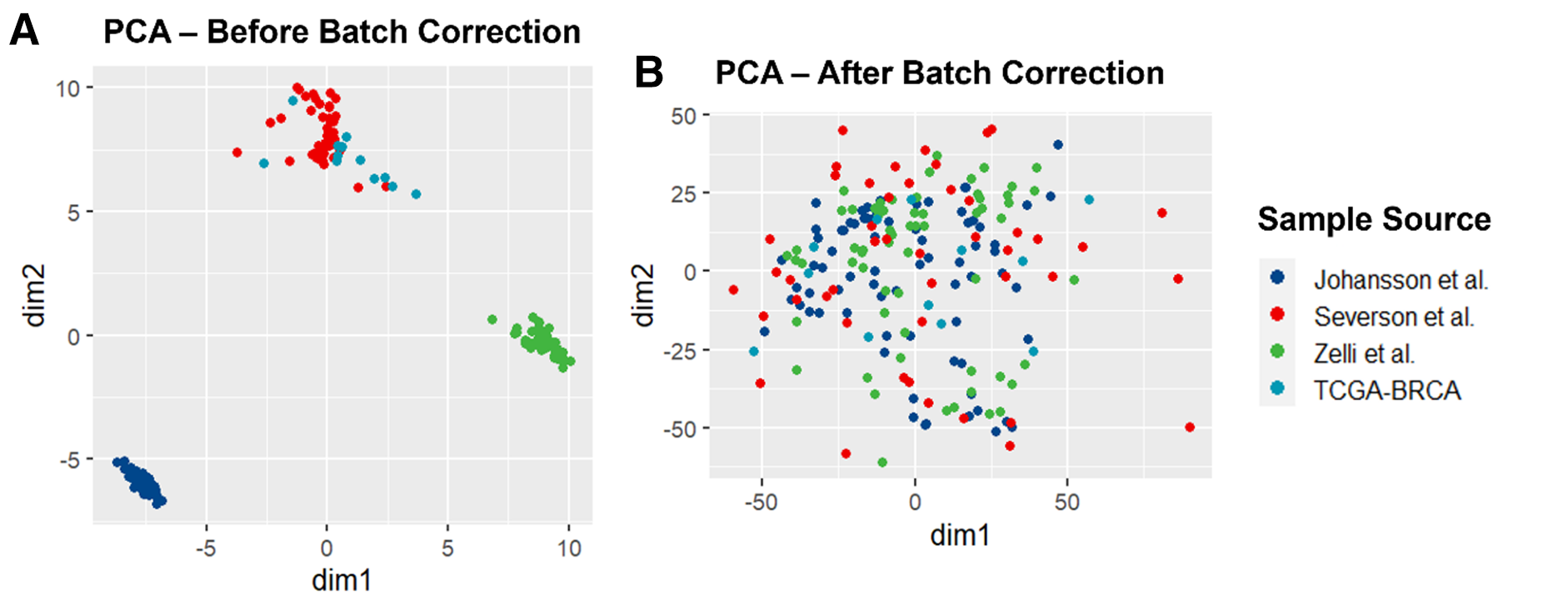


**Figure S1.** Confirmation of batch correction by principal component analysis (PCA): PCA plots of cases annotated according to their source dataset before (A) and after (B) batch correction. The plot in (B) confirms minimisation of batch effects.


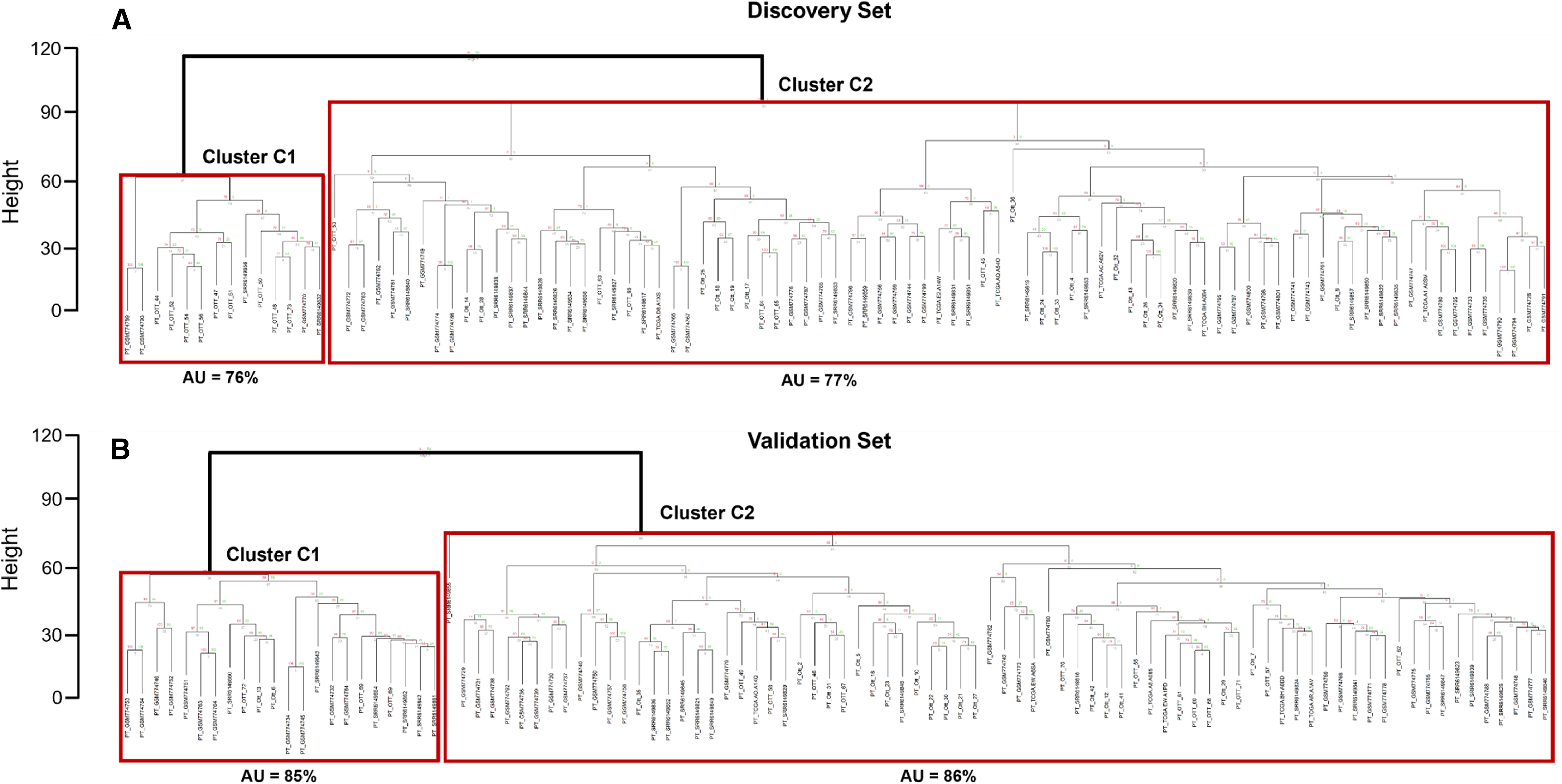


**Figure S2.** Identification of two stable clusters in the discovery and validation sets: Two stable clusters (highlighted in red) were identified in both the (A) discovery (AU probability of 76% and 77% for Clusters C1 and C2, respectively) and (B) validation set (AU probability of 85% and 86% for Clusters C1 and C2, respectively).


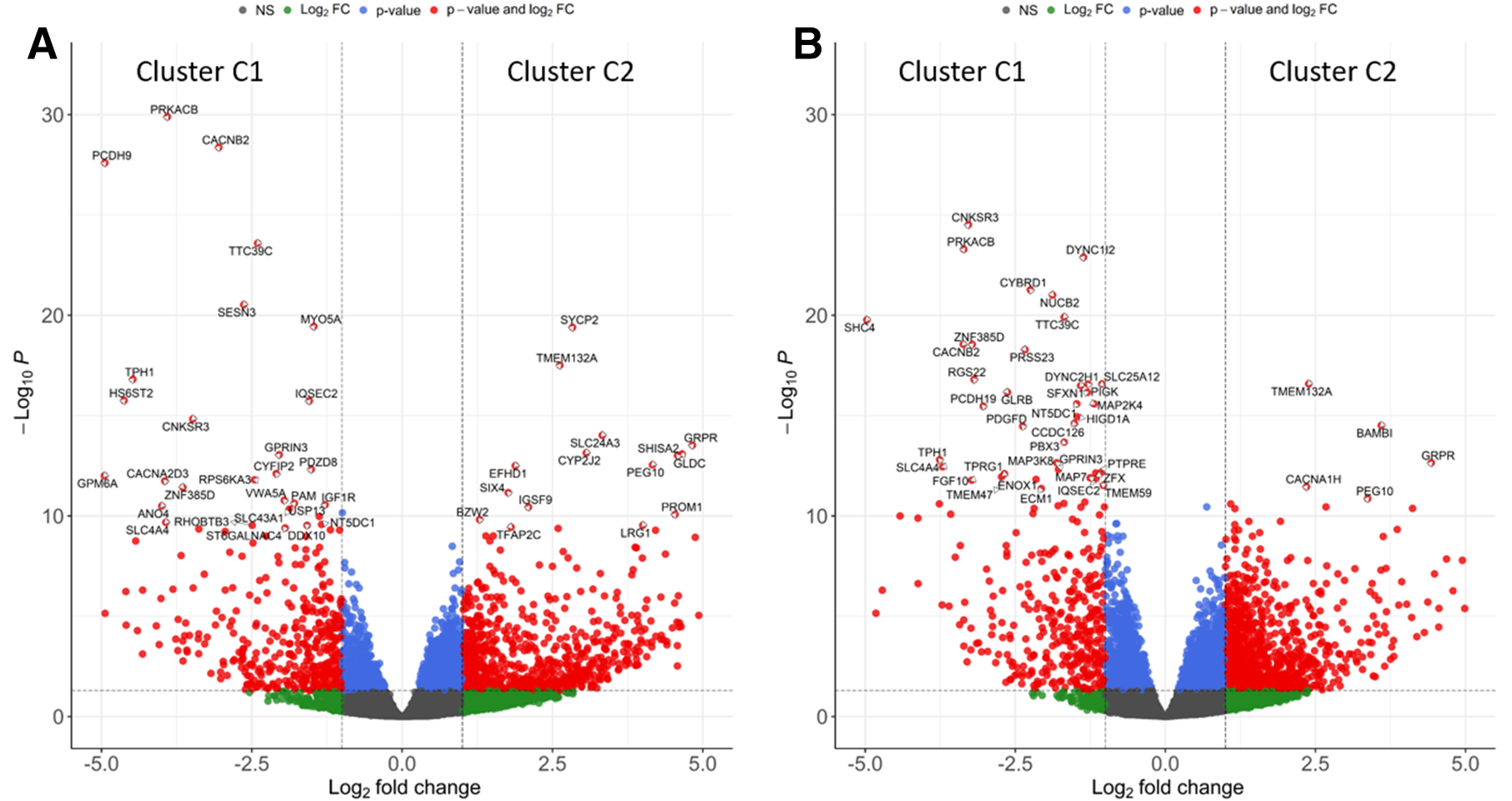


**Figure S3.** Volcano plots of DEGs in Clusters C1 and C2 in the discovery and validation sets: Discovery (A) and validation (B) sets are shown. Genes showing greatest variability and with the lowest adjusted *p* values are labelled.


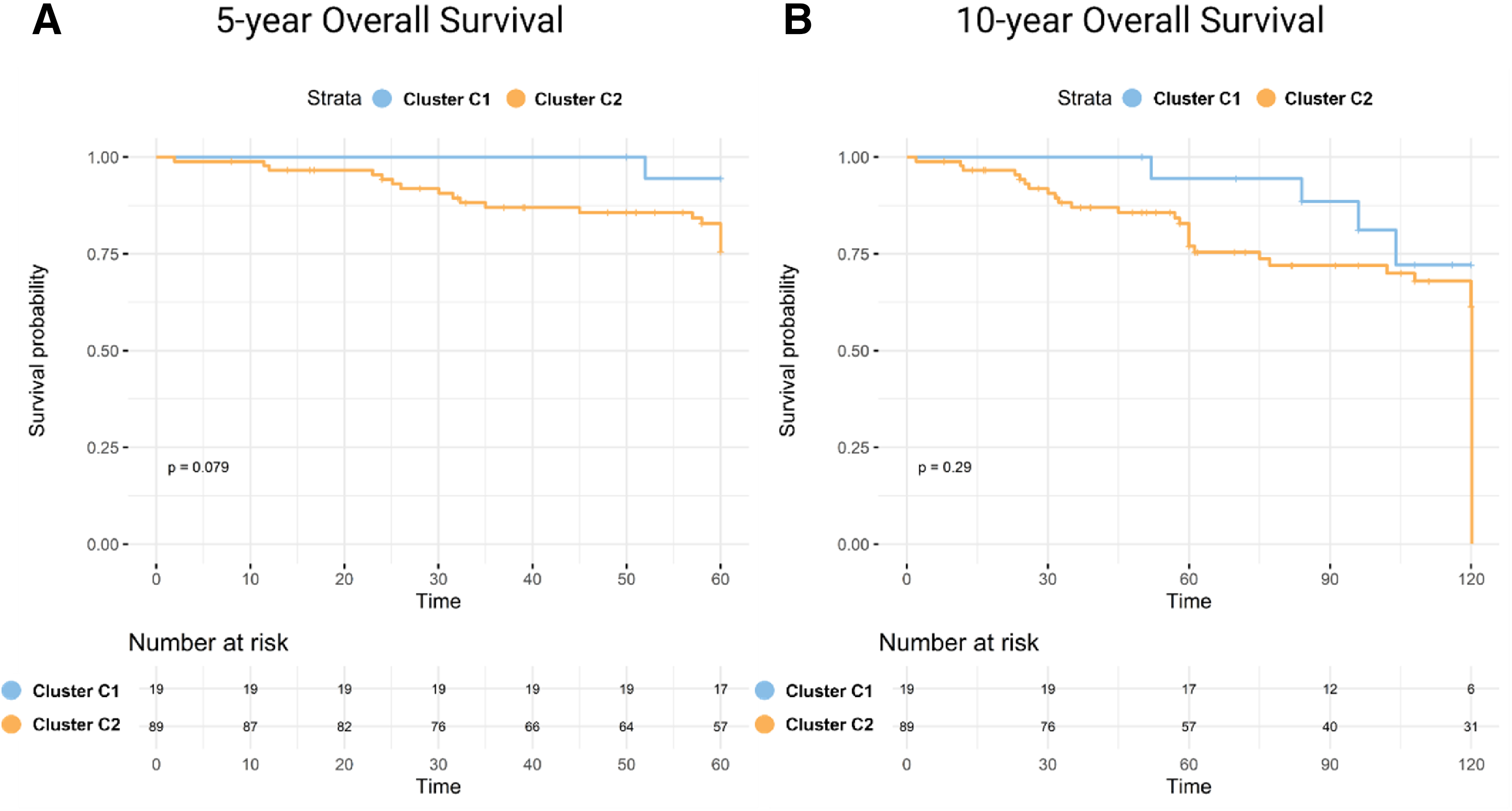


**Figure S4.** Kaplan–Meier curves comparing OS from cases in Clusters C1 and C2. Kaplan–Meier curves comparing OS between patients belonging to Clusters C1 and C2 at (A) 5 years and (B) 10 years. Survival time is shown in months.


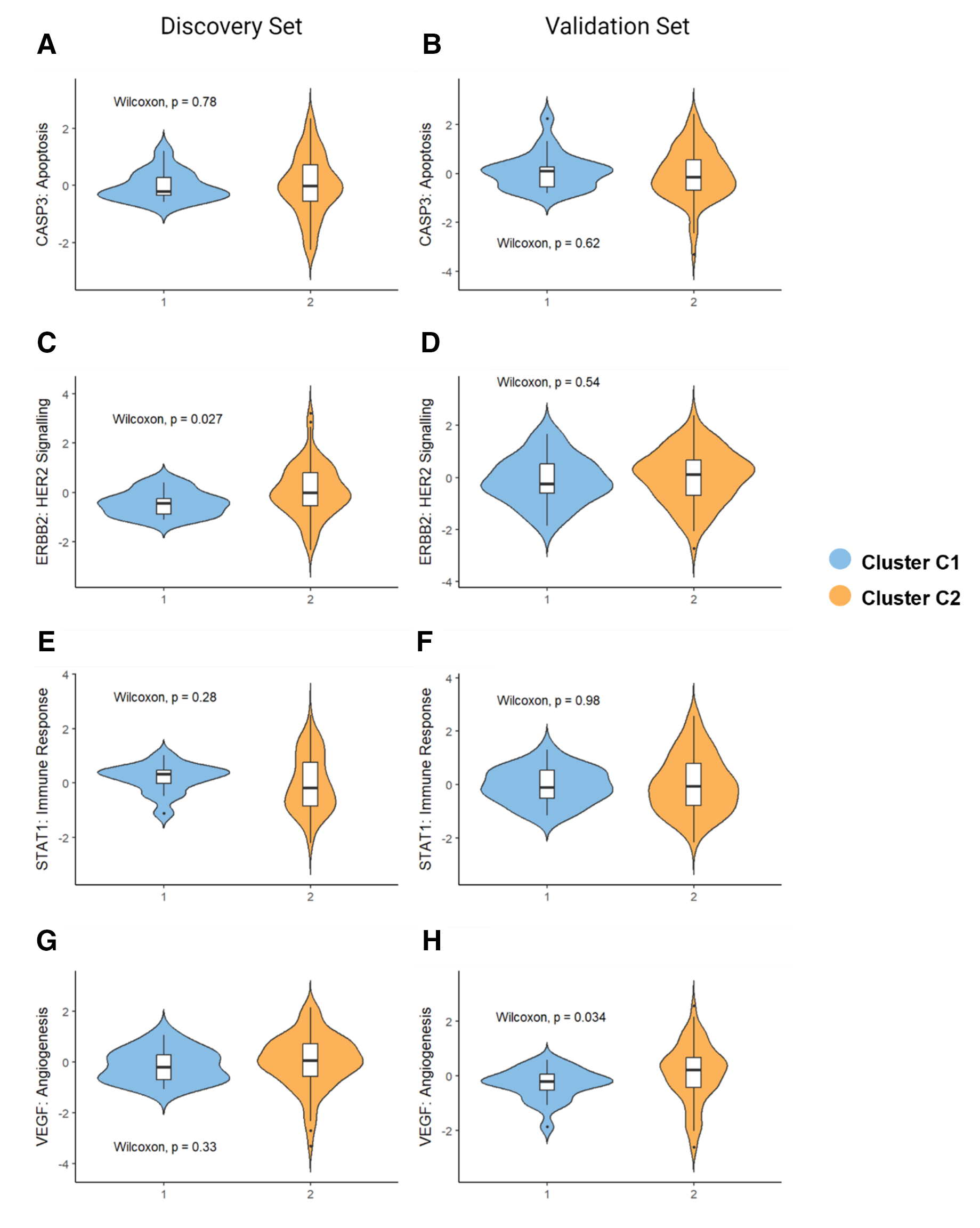


**Figure S5.** Distribution of GEX module scores that were similar between clusters or had dissimilar results between the discovery and validation sets. Violin plots showing GEX modules that either had similar scores between Clusters C1 and C2 or had significantly different scores in either the (i) discovery or (ii) validation set. GEX modules shown are (A and B) apoptosis (*CASP3* signature), (C and D) HER2 signalling (*ERBB2* signature), (E and F) immune response (*STAT1* signature), and (G and H) angiogenesis (*VEGF* signature) modules.


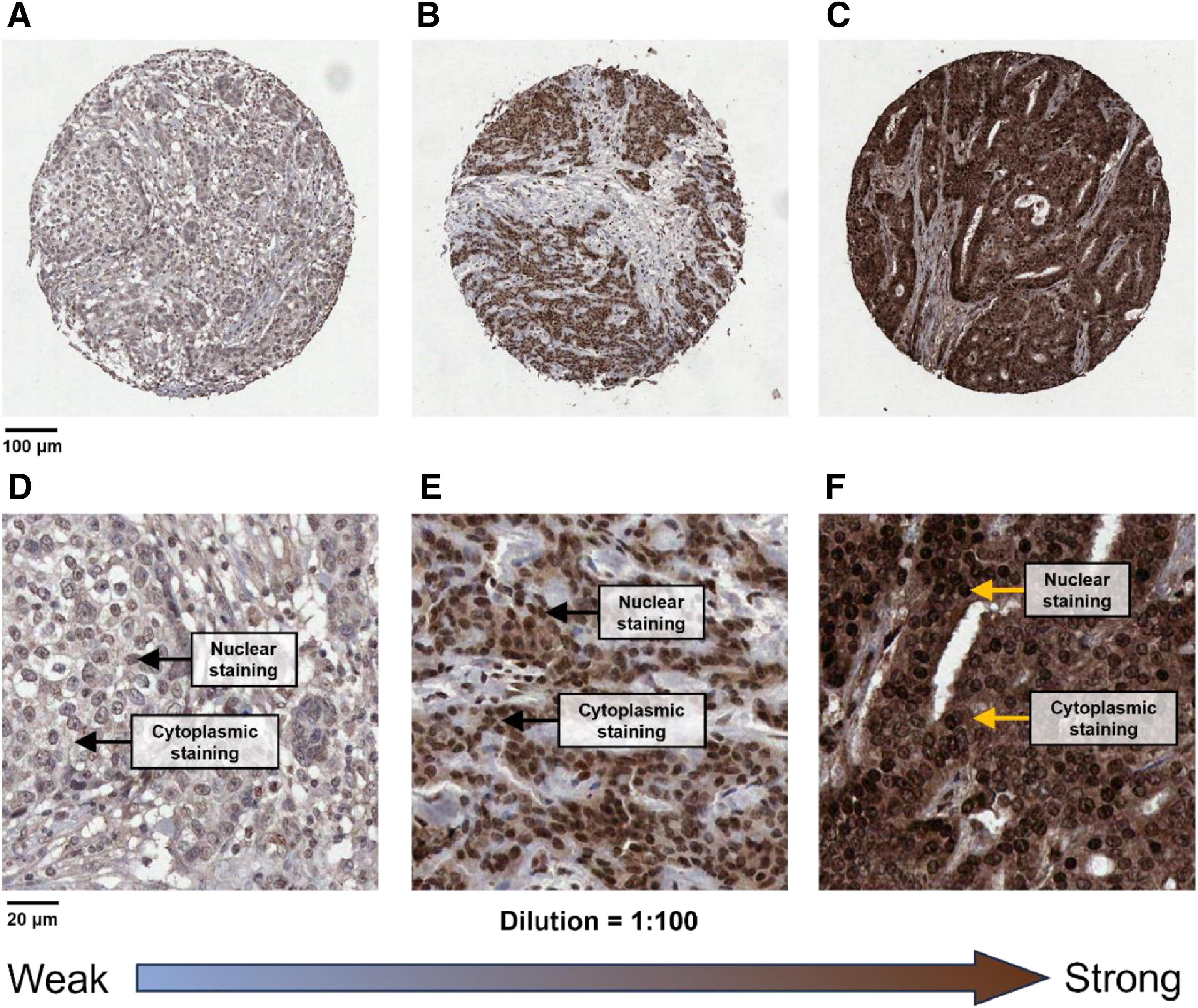


**Figure S6.** Staining characteristics of S104 in male BC TMAs. Representative TMA cores showing staining intensities and nuclear and cytoplasmic subcellular localisation (A–C) at 5× magnification (scale bar, 100 μm) and (D–F) at 20× magnification (scale bar, 20 μm). Primary antibody dilution was 1:100.


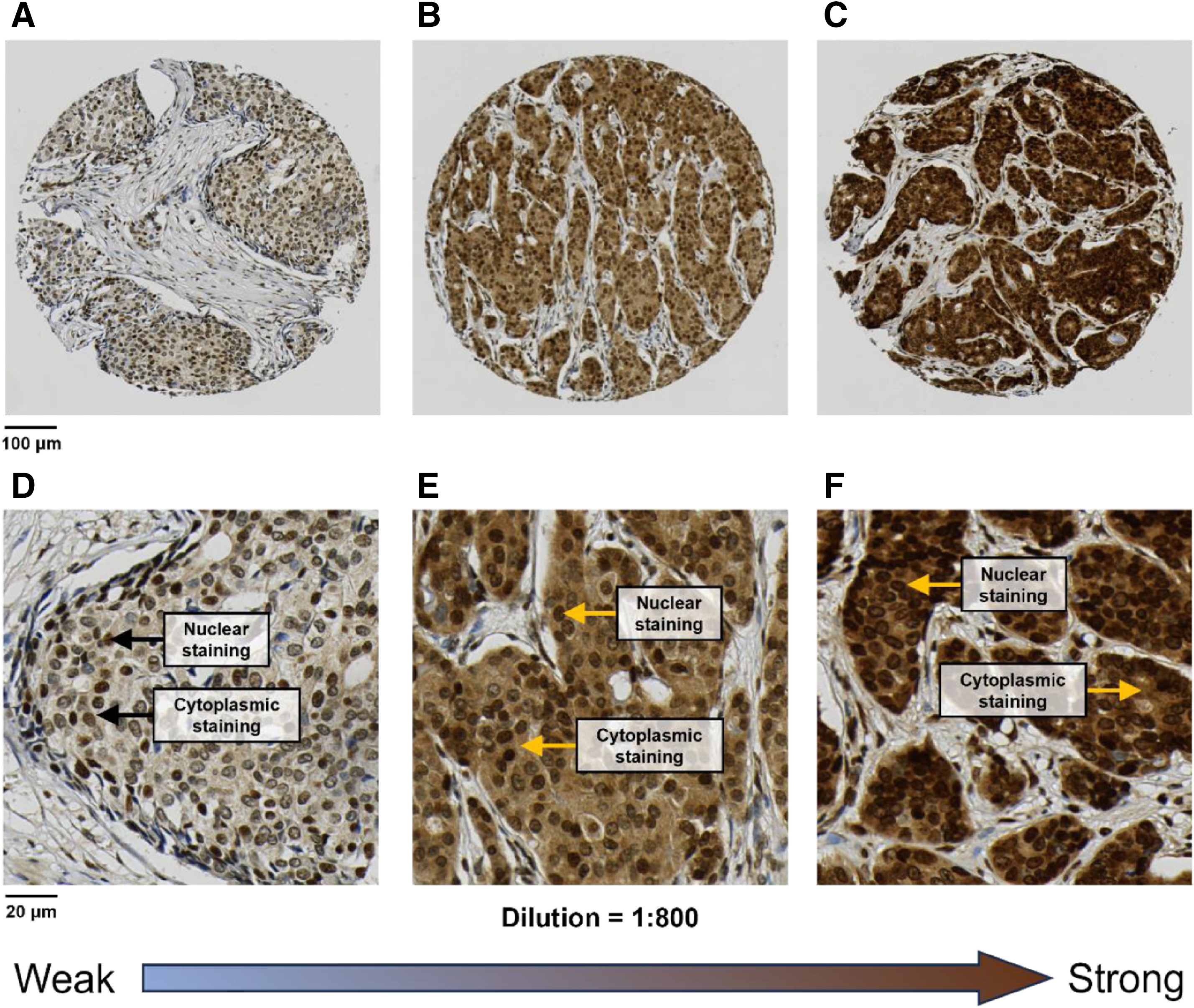


**Figure S7.** Staining characteristics of S118 in male BC TMAs. Representative TMA cores showing staining intensities and nuclear and cytoplasmic subcellular localisation (A–C) at 5× magnification (scale bar, 100 μm) and (D–F) at 20× magnification (scale bar, 20 μm). Primary antibody dilution was 1:800.


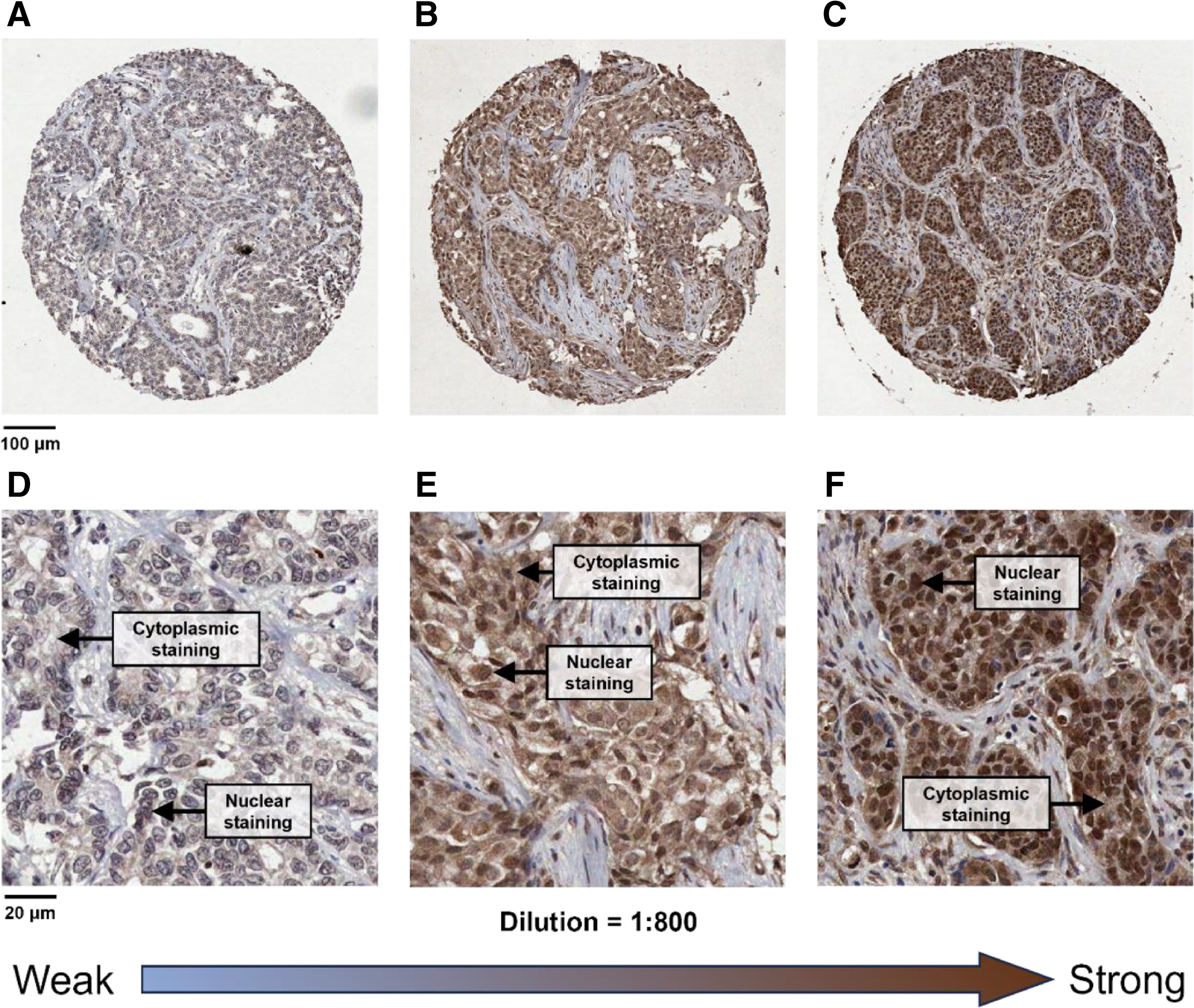


**Figure S8.** Staining characteristics of S167 in male BC TMAs. Representative TMA cores showing staining intensities and nuclear and cytoplasmic subcellular localisation (A–C) at 5× magnification (scale bar, 100 μm) and (D–F) at 20× magnification (scale bar, 20 μm). Primary antibody dilution was 1:800.


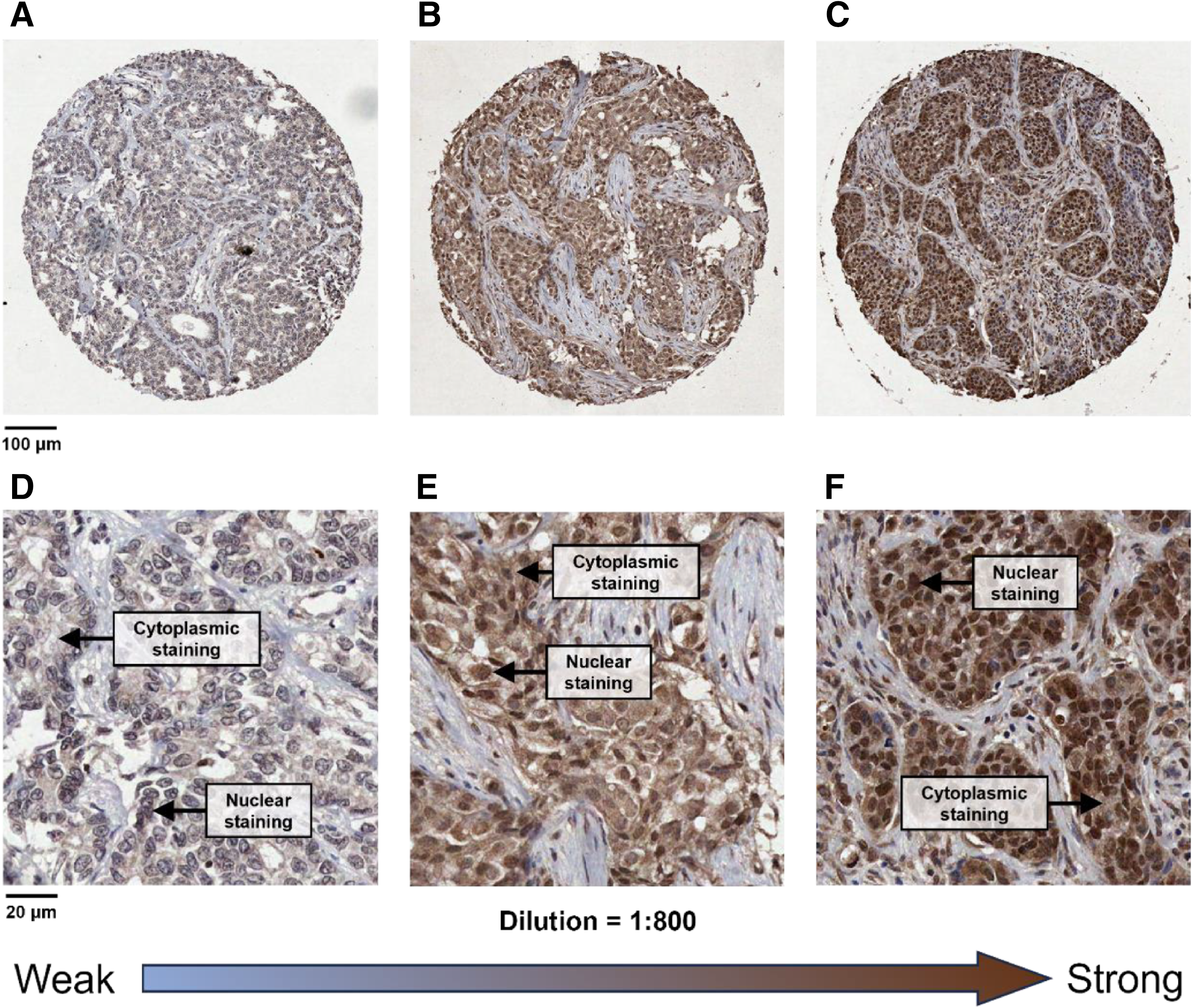


**Figure S9.** Staining characteristics of S294 in male BC TMAs. Representative TMA cores showing the various staining intensities and subcellular localisation (nuclear and cytoplasmic) of pER-S294 with weaker to stronger staining shown in (A–C) at 5× magnification and (D–F) at 20× magnification.

**Supplementary Tables**

**Table S1.** Male BC datasets used for integrated bioinformatics

| **Dataset (*n*)** | **Accession number** | **Format** | **Clinical information** |
| --- | --- | --- | --- |
| Johansson (74) [16] | NCBI-GEO GSE31259 | log2 (raw counts) transformed format. Raw count data obtained by reversing the log2 transformation. | Available in NCBI-GEO |
| Severson (46) [24] | NCBI-GEO GSE104730 | rlog transformed format. FASTQ files downloaded for each case and aligned to the reference human genome (Ensembl GRCh38.p14) with STAR (v2.7.11) [31] using default parameters. Raw counts were obtained using HTSeq (2.0.4) [32] carried out in Python 3.11. | Provided by Prof Paul van Diest |
| Zelli (63)* [18] | n/a | Raw counts. Further pre-processing not required. | Provided by Prof Laura Ottini |
| TGCA (12) [21] | n/a | Raw counts data in TCGA-BRCA dataset downloaded individually from the [Genomic Data Commons Data Portal](https://portal.gdc.cancer.gov/) and combined into a dataset | Downloaded from [cBioPortal for Cancer Genomics](https://www.cbioportal.org/) |

*Cases had germline mutations in relevant BC susceptibility genes: *BRCA2*, *BRCA1*, *PALB2*, *RAD50* and *RAD51D*

**Table S2.** Sample identifiers for each patient in datasets obtained from NCBI GEO and TCGA

| **Dataset: GSE104730* (*n* = 46)** | | **Dataset: GSE31259 (*n* = 74)** | **Dataset: TCGA-BRCA (*n* = 12)** |
| --- | --- | --- | --- |
| **Sample ID** | **Run ID** | **Sample ID** | **Sample ID** |
| GSM2806798 | SRR6149817 | GSM774728 | TCGA-A1-A0SM |
| GSM2806799 | SRR6149818 | GSM774729 | TCGA-A8-A085 |
| GSM2806800 | SRR6149819 | GSM774730 | TCGA-AC-A62V |
| GSM2806801 | SRR6149820 | GSM774731 | TCGA-AO-A1KQ |
| GSM2806802 | SRR6149821 | GSM774732 | TCGA-AQ-A54O |
| GSM2806803 | SRR6149822 | GSM774733 | TCGA-AR-A1AV |
| GSM2806804 | SRR6149823 | GSM774734 | TCGA-BH-A0B4 |
| GSM2806805 | SRR6149824 | GSM774735 | TCGA-BH-A0DD |
| GSM2806806 | SRR6149825 | GSM774736 | TCGA-D8-A1XS |
| GSM2806807 | SRR6149826 | GSM774737 | TCGA-E2-A14W |
| GSM2806808 | SRR6149827 | GSM774738 | TCGA-EW-A1PD |
| GSM2806809 | SRR6149828 | GSM774739 | TCGA-EW-A6SA |
| GSM2806810 | SRR6149829 | GSM774740 |  |
| GSM2806811 | SRR6149830 | GSM774741 |  |
| GSM2806812 | SRR6149831 | GSM774742 |  |
| GSM2806813 | SRR6149832 | GSM774743 |  |
| GSM2806814 | SRR6149833 | GSM774744 |  |
| GSM2806815 | SRR6149834 | GSM774745 |  |
| GSM2806816 | SRR6149835 | GSM774746 |  |
| GSM2806817 | SRR6149836 | GSM774747 |  |
| GSM2806818 | SRR6149837 | GSM774748 |  |
| GSM2806819 | SRR6149838 | GSM774749 |  |
| GSM2806820 | SRR6149839 | GSM774750 |  |
| GSM2806821 | SRR6149840 | GSM774751 |  |
| GSM2806822 | SRR6149841 | GSM774752 |  |
| GSM2806823 | SRR6149842 | GSM774753 |  |
| GSM2806824 | SRR6149843 | GSM774754 |  |
| GSM2806825 | SRR6149844 | GSM774755 |  |
| GSM2806826 | SRR6149845 | GSM774756 |  |
| GSM2806827 | SRR6149846 | GSM774757 |  |
| GSM2806828 | SRR6149847 | GSM774758 |  |
| GSM2806829 | SRR6149848 | GSM774759 |  |
| GSM2806830 | SRR6149849 | GSM774760 |  |
| GSM2806831 | SRR6149850 | GSM774761 |  |
| GSM2806832 | SRR6149851 | GSM774762 |  |
| GSM2806833 | SRR6149852 | GSM774763 |  |
| GSM2806834 | SRR6149853 | GSM774764 |  |
| GSM2806835 | SRR6149854 | GSM774765 |  |
| GSM2806836 | SRR6149855 | GSM774766 |  |
| GSM2806837 | SRR6149856 | GSM774767 |  |
| GSM2806838 | SRR6149857 | GSM774768 |  |
| GSM2806839 | SRR6149858 | GSM774769 |  |
| GSM2806840 | SRR6149859 | GSM774770 |  |
| GSM2806841 | SRR6149860 | GSM774771 |  |
| GSM2806842 | SRR6149861 | GSM774772 |  |
| GSM2806843 | SRR6149862 | GSM774773 |  |
|  |  | GSM774774 |  |
|  |  | GSM774775 |  |
|  |  | GSM774776 |  |
|  |  | GSM774777 |  |
| **Sample ID** | **Run ID** | **Sample ID** | **Sample ID** |
|  |  | GSM774778 |  |
|  |  | GSM774779 |  |
|  |  | GSM774780 |  |
|  |  | GSM774781 |  |
|  |  | GSM774782 |  |
|  |  | GSM774783 |  |
|  |  | GSM774784 |  |
|  |  | GSM774785 |  |
|  |  | GSM774786 |  |
|  |  | GSM774787 |  |
|  |  | GSM774788 |  |
|  |  | GSM774789 |  |
|  |  | GSM774790 |  |
|  |  | GSM774791 |  |
|  |  | GSM774792 |  |
|  |  | GSM774793 |  |
|  |  | GSM774794 |  |
|  |  | GSM774795 |  |
|  |  | GSM774796 |  |
|  |  | GSM774797 |  |
|  |  | GSM774798 |  |
|  |  | GSM774799 |  |
|  |  | GSM774800 |  |
|  |  | GSM774801 |  |

*For dataset GSE104730, sequencing run IDs were used as sample identifiers due to end-to-end processing from raw FASTQ files, where each run corresponds uniquely to a single patient. Run ID to sample ID mapping is provided. For datasets GSE31259 and TCGA-BRCA, run IDs were not used as end-to-end processing was not required. Detailed processing steps for each dataset is given in Supplementary Methods.

**Table S3.** Clinicopathological characteristics of the male BC patient cohort used for IHC

| **Characteristic** | **Status** | ***n* (%)** |
| --- | --- | --- |
| Age | < 65 years | 183 (43.7) |
|  | < 65 years | 204 (52.7) |
| Grade | 1 | 50 (11.6%) |
|  | 2 | 210 (48.6%) |
|  | 3 | 172 (39.8%) |
| Node status | Positive | 168 (52%) |
|  | Negative | 155 (48%) |
| Histological type | Ductal | 370 (84.3%) |
|  | Lobular | 3 (0.7%) |
|  | Papillary | 16 (3.6%) |
|  | Micropapillary | 3 (0.7%) |
|  | Intraductal papillary | 6 (1.4%) |
|  | Mucinous | 12 (2.7%) |
|  | Adenocarcinoma | 11 (2.5%) |
|  | Mixed* | 14 (3.2%) |
|  | Other** | 4 (0.9%) |
| ERα | Positive | 440 (93.6%) |
|  | Negative | 30 (6.4%) |
| PR | Positive | 383 (83.1%) |
|  | Negative | 78 (16.9%) |
| AR | Positive | 273 (68.3%) |
|  | Negative | 127 (31.7%) |

*Includes ductal + cribriform (*n* = 1), ductal + papillary (*n* = 1), ductal + micropapillary (*n* = 2), and unspecified mixed type (*n* = 10). **Includes cribriform (*n* = 2), medullary (*n* = 1), and tubular type (*n* =1)

**Table S4** Details of phosphorylated antibodies

| **Antibody (type)** | **Supplier**  **(catalogue number)** | **Dilution** | **Retrieval** | **Positive control** |
| --- | --- | --- | --- | --- |
| S104 (rabbit polyclonal) | Abcam (ab30656) | 1:100 | HIER^1^, pH 6 | Female BC |
| S118 (rabbit monoclonal) | Abcam (ab32396) | 1:800 | HIER, pH 6 | Female BC |
| S167 (rabbit polyclonal) | Abcam (ab131105) | 1:100 | HIER, pH 6 | Female BC |
| S294 (rabbit monoclonal) | Abcam (ab207602) | 1:800 | HIER, pH 6 | Female BC |

^1^Heat induced epitope retrieval

**Table S5.** Breakdown of cases based on source dataset and their association with Clusters C1 and C2 in the discovery and validation sets

| **Source Dataset** | **Discovery set** | | | **Validation set** | | |
| --- | --- | --- | --- | --- | --- | --- |
|  | **Cluster C1** | **Cluster C2** | **p-value (Fisher’s exact test)** | **Cluster C1** | **Cluster C2** | **p-value (Fisher’s exact test)** |
| Johansson *et al* [17] | 3 (3.1%) | 34 (35.1%) | *0.07* | 11 (11.2%) | 26 (26.5%) | 0.27 |
| Severson *et al* [24] | 2 (2.1%) | 22 (22.7%) |  | 6 (6.1%) | 16 (16.3%) |  |
| Zelli *et al* [18] | 9 (9.3%) | 21 (21.7%) |  | 5 (5.1%) | 28 (28.6%) |  |
| TCGA-BRCA [21] | 0 (0%) | 6 (6.2%) |  | 0 (0%) | 6 (6.1%) |  |

**Table S6.** Breakdown of cases based on predicted PAM50 subtype and the association with Clusters C1 and C2 in the discovery and validation sets

| **Predicted PAM50 subtype** | **Discovery set** | | | **Validation set** | | |
| --- | --- | --- | --- | --- | --- | --- |
|  | **Cluster C1** | **Cluster C2** | **p-value (Fisher’s exact test)** | **Cluster C1** | **Cluster C2** | **p-value (Fisher’s exact test)** |
| Luminal A | 12 (12.4%) | 22 (22.7%) | *0.002**  Adjusted *p*-value = *0.009** | 12 (12.2%) | 17 (17.3%) | *0.005**  Adjusted *p*-value = *0.01** |
| Luminal B | 2 (2.1%) | 39 (40.2%) |  | 8 (8.2%) | 31 (31.6%) |  |
| HER2-enriched | 0 (0%) | 11 (11.3%) |  | 0 (0%) | 18 (18.4%) |  |
| Basal-like | 0 (0%) | 7 (7.2%) |  | 0 (0%) | 6 (6.1%) |  |
| Normal-like | 0 (0%) | 4 (4.1%) |  | 2 (20%) | 4 (4.1%) |  |
| Total | *n* = 97 | |  | *n* = 98 | |  |

**Table S7.** Breakdown of cases based on their hormone receptor profiles and the estimated PAM50 subtypes in the discovery and validation sets

| **Hormone Receptor Status** | **Discovery Set** | | | | | | **Validation Set** | | | | | |
| --- | --- | --- | --- | --- | --- | --- | --- | --- | --- | --- | --- | --- |
|  | **Predicted PAM50 Status** | | | | | ***p*-value (Fisher’s exact test)** | **Predicted PAM50 Status** | | | | | ***p*-value (Fisher’s exact test)** |
|  | **Luminal A** | **Luminal B** | **HER2-Enriched** | **Basal-like** | **Normal-like** |  | **Luminal A** | **Luminal B** | **HER2-Enriched** | **Basal-like** | **Normal-like** |  |
| ERα+/PR+ | 31 (35.2%) | 32 (36.4%) | 7 (7.9%) | 6 (6.8%) | 2 (2.3%) | 0.17 | 26 (28.9%) | 29 (32.2%) | 11 (12.2%) | 2 (2.2%) | 5 (5.6%) | 0.22 |
| ERα+/PR- | 1 (1.1%) | 5 (5.7%) | 1 (1.1%) | 0 (0%) | 0 (0%) |  | 2 (2.2%) | 5 (5.6%) | 4 (4.4%) | 2 (2.2%) | 0 (0%) |  |
| ERα-/PR+ | 0 (0%) | 1 (1.1%) | 0 (0%) | 0 (0%) | 0 (0%) |  | 1 (1.1%) | 0 (0%) | 1 (1.1%) | 0 (0%) | 0 (0%) |  |
| ERα-/PR- | 0 (0%) | 0 (0%) | 1 (1.1%) | 1 (1.1%) | 0 (0%) |  | 0 (0%) | 1 (1.1%) | 1 (1.1%) | 0 (0%) | 0 (0%) |  |
| Cases with available ERα/PR status* | *n* = 88 | | | | |  | *n* = 90 | | | | |  |

*ERα/PR status was unavailable for 9 cases in the discovery cohort and 8 in the validation
